# A Practical Treatise on Genitourinary and Venereal Diseases and Syphilis

**Published:** 1904-11

**Authors:** 


A Practical Treatise on Genitourinary and Venereal Diseases and Syphilis. By Robert W. Taylor, A.M., M.D., Clinical Professor of Genitourinary Diseases, College of Physicians and Surgeons (Columbia University), New York. Third edition, thoroughly revised. Octavo, pp. 757. With 163 illustrations and 39 plates in colors and monochrome. New York and Philadelphia: Lea Brothers & Company. 1904. (Price, cloth, $5.00; leather, $6.00; half morocco, $6.50 net.)

The rapidity with which the second edition of this work was exhausted was not alone gratifying to the author and publisher, but necessitated the issue of a third edition which gave Taylor a chance to go over his book and rather broaden its scope in certain sections. One of the most striking features of the author's literary work is that he steers clear of the overelaboration of rare anomalies, malformations and unimportant diseases.

They are mentioned of course as a matter of curiosity apparently, as they should be, but being rare they are of little benefit to the everyday practitioner whose labors are rather more concerned with the daily genitourinary affections and not with the curiosities. The book is largely made up of trustworthy practical information omitting surgical and therapeutic procedures of doubtful value. All the new genitourinary operations are given a place, with description and opinion as to their availability in certain cases. An air of solid and confidential conservatism marks the whole book. In dealing with gonorrhea Taylor enters an emphatic protest against the fallacies and dangers of the views of some of the latter day faddists who fly the green flag of fanaticism and fairly wallow in the glory of the miraculous cures of their newly discovered remedies. Yet even in the third edition the author has not quite reached the point where he can conscientiously condemn the irrigation method of treatment of gonorrhea, although he speaks of it with veiled dislike and without any positive commendation or condemnation, rather tending to the use of the reflux catheter. It is when he reaches the consideration of the use of silver products in gonorrhea that he gives rein to his fancy and speaks without the slightest hesitation or restraint. With the preliminary statement that silver nitrate is by all odds the best form of silver to use, best because it gives best results and is not masquerading under a fanciful name, he takes up the various patent preparations now on the market for which so many really miraculous cures have been claimed. Some of these he places among the remedies which have been discarded; others he classes among the therapeutic curiosities; while of still another and later preparation, probably the most brilliantly advertised special silver product now on the market, he says it has given no results in his clinic which have not been achieved by the use of a weak solution of silver nitrate. The whole book is marked with that solid common sense which begets a feeling of safety, confidence, and certainty in the treatment of disease.

Syphilis is dealt with in the same intelligent way, which marked the preparation of this subject in previous editions of the book. There are 25 new illustrations and 12 plates in color and monochrome largely made from the author's own cases. An Italian edition of the book has recently been issued.

